# Herbal Formula Fo Shou San Attenuates Alzheimer's Disease-Related Pathologies via the Gut-Liver-Brain Axis in APP/PS1 Mouse Model of Alzheimer's Disease

**DOI:** 10.1155/2019/8302950

**Published:** 2019-06-17

**Authors:** Jia Lu, Pengfei Guo, Xiaoqiu Liu, Yongbin Zhang, XueJun Guo, Xin Gao, Yuan Chen

**Affiliations:** ^1^Piwei Institute, Guangzhou University of Chinese Medicine, Guangzhou, Guangdong 510405, China; ^2^Experimental Animal Center, Guangzhou University of Chinese Medicine, Guangzhou, Guangdong 510405, China

## Abstract

Fo Shou San (FSS) is an ancient paired-herb decoction, used in China to treat blood deficiency, blood stasis, stroke, and ischemic cerebral vascular disease for about one thousand years. The mechanisms associated with these properties, however, are not completely understood. Gut bacteria, gut bacterial lipopolysaccharides (LPS), alkaline phosphatase (AP), and lipid peroxidation are common biochemical signaling that takes place on gut-liver-brain axis. Growing evidences have revealed that gut bacterial lipopolysaccharides (LPS) enter the systemic circulation via the portal vein, and finally entering the brain tissue is an important cause of inflammatory degeneration of Alzheimer's disease (AD). Alkaline phosphatase (AP) dephosphorylates LPS forming a nontoxic LPS and reduces systemic inflammation via gut-liver-brain axis. In this study, to identify the differentially gut-liver-brain axis among APP/PS1 mice, FSS-treated APP/PS1 mice, and control mice, behavioral tests were performed to assess the cognitive ability and hematoxylin-eosin staining was used to assess neuronal damage in the hippocampus; immunohistochemistry, western blotting, a quantitative chromogenic end-point Tachypleus amebocyte lysate (TAL) assay kit, Malondialdehyde (MDA) assay kit, AP Assay Kit, and real-time quantitative PCR (qPCR) were used to assess the level of LPS, MDA, AP, and gut bacteria. We found that FSS regulates gut-liver-brain axis to regulate AP and gut bacteria and attenuate the LPS-related systemic inflammation, oxidative stress (MDA), and thereby AD-related pathology in APP/PS1 mice. This is the first study to provide a reference for FSS-treated AD mice to aid in understanding the underlying mechanisms of FSS. FSS may also improve gastrointestinal tract barrier and blood-brain barrier and thus ameliorates the symptoms of AD; this is subject to our further study.

## 1. Introduction

Alzheimer's disease (AD) is a neurodegenerative disorder characterized by progressive memory decline and subsequent broader loss of cognitive function [1]. These changes lead to a decline in the quality of life of AD patients, and an increase in the number of AD patients will be a serious public health challenge. Current AD drugs target cholinergic and glutamatergic neurotransmission, thus ameliorating symptoms; however, the long-term efficacy of these drugs remains controversial in clinical practice [2, 3]. Moreover, there is still no effective intervention to prevent, halt, or reverse AD. Because they can treat multiple targets and are safe and suitable for long-term use, traditional Chinese herbs and formulas such as Bushen-Yizhi Formula [4], Herbal Formula Tian-Ma-Gou-Teng-Yin [5], Dang gui shao yao san, Chai hu jia long gu mu li tang [6], and more than 10 other herbs exhibiting significant effects on AD have become more and more frequently used as treatments for the disease [7]. Fo Shou San (FSS) is an ancient paired-herb decoction composed of* Angelica sinensis* radix (Danggui) and* Ligusticum chuanxiong* Hort rhizome (Chuangxiong) in a weight ratio of 3:2. Use of FSS was first recorded by Xu Shuwei in Puji Benshi, published during the Song Dynasty (AD 1132) in China and has been used to treat patients with blood deficiency, blood stasis, stroke, and ischemic cerebral vascular disease for about a 1000 years. FSS has been shown to improve hemorheology, enrich blood, and alleviate disturbed metabolic pathways in acute blood stasis model rats, as well as inhibit inflammation and lipid peroxidation [8–12]. However, whether FSS is effective in treating (AD) patients has not been tested previously. Amyloid-*β* (A*β*) and hyperphosphorylated Tau protein, along with synaptic and neuronal losses, are the main features of AD [13]. However, the pathogenesis of AD is still unclear. Gut bacteria, gut bacterial lipopolysaccharides (LPS), alkaline phosphatase (AP), and lipid peroxidation are common biochemical features involved in signaling in the gut-liver-brain axis [14–21]. Growing evidence has revealed that gut bacterial LPS enter the systemic circulation via the liver portal circulation and become important contributors to inflammatory degeneration in AD [22, 23]. Based on this information, in the present study we investigated differences in gut bacteria, AP, LPS, and lipid peroxidation (malondialdehyde, MDA) in the gut, liver, serum, and brain between APP/PS1 AD mice, FSS-treated APP/PS1 AD mice, and control mice to determine whether FSS exerts any protective effects in APP/PS1 AD mice, and if so, by what specific mechanism(s).

## 2. Materials and Methods

### 2.1. Animal Part

#### 2.1.1. Animals

Mice were maintained under the specific pathogen-free standard living conditions of the Animal Center of Guangzhou University of Chinese Medicine, namely, room temperature (RT) at 22 ± 1°C, humidity at 55 ± 5%, and a 12-h light/12-h dark cycle. The mice were separately given water and pellet food* ad libitum*. The AD model APP/PS1 is based on C57BL/6J mice and carries the equivalents of the human APP Swedish and human PS1 deltaE9 mutations. Moreover, this model begins to show learning and memory defects at 3 months of age, and senile plaques begin to appear at 4 months. At 12 months of age, a considerable number of senile plaques are present, and the mice have a pathological phenotype similar to AD.

#### 2.1.2. Preparation of FSS

FSS consists of a mixture of two medicinal plants (Table 1). The herbs we used were purchased from the First Affiliated Hospital of Guangzhou University of Chinese Medicine. We prepared the FSS according to the standards described in Pharmacopoeia of the People's Republic of China (2010 Edition). Briefly, the decoction of herbs and water at a ratio of 1:8 was boiled for 2 hours for the first extraction, and then 6 times the amount of water was added and boiled for 2 hours for a second extraction. Both decoctions were collected and concentrated to a final density of 0.64g/ml and were stored initially at 4°C and then at −20°C. Before use, the concentrate was removed from the freezer, thawed, and diluted with distilled water to the required concentration. Putative active substances in the FSS concentrate were detected by direct analysis in real time (DART) of flight mass spectrometry (TOFMS) carried out at ICAS Analysis Technology Service Co., Ltd. (Shanghai, China).

#### 2.1.3. Administration of FSS

Six-month-old male APP/PS1 mice were randomly divided into a model group (AD group); high-, medium-, and low-dose FSS APP/PS1 groups (FFS-H, FFS-M, and FFS-L; doses of 6.4, 3.2, and 1.6 g/kg/day, respectively) and a positive control group (dose of 2 mg/kg/day donepezil). Age-matched C57BL/6J mice as the wild type (WT) control group and the WT group and AD group were given an equal volume of ultrapure water. All treatments were administered intragastrically once a day for 8 weeks. After administration, Morris water maze, Y maze, and novel object recognition tests were used to assess cognitive function.

#### 2.1.4. Morris Water Maze Tests

Morris water maze tests were used to evaluate spatial learning and memory [24]. The water maze equipment (Shanghai XinRuan Information Technology Co., Ltd., Shanghai, China) comprises a circular pool, a circular hidden escape platform, and a recording system. The pool, filled with water at 24°C, was spatially divided into four imaginary quadrants (first, second, third, and fourth) by a computerized tracking/image analyzer system. The circular escape platform (10 cm in diameter), which was located in the center of the third quadrant and served as the target quadrant of the pool, was 1-2 cm below the white opaque water surface. The tests were performed in a dark room. Mice were trained before being given 6 consecutive days of orientation and navigation tests. Each daily training session consisted of four sequential training trials in which the location at which the mouse was dropped into the pool changed randomly. Each trial began with the mouse placed facing towards the wall into the pool, and then the recording system was started to record the time. Escape latency and swim path tracking were recorded on video tape until the mouse landed on the platform for 2 s. If the mouse failed to locate the platform within 60 s, it was guided to the platform and kept there for 15 s. On the last day of the probe trials, each mouse was allowed to swim freely in the pool for 60 s without a platform and the number of times it swam to the original platform position, the time it spent in the third quadrant, and its swimming speed were recorded.

#### 2.1.5. Y Maze Tests

Y maze tests were used to analyze the short-term spatial memory and exploratory activity of the experimental mice [24]. The Y maze is a horizontal maze (40 cm long and 10 cm wide with 12-cm-high walls) with three arms that converge at equal angles. Each mouse was placed at the end of one arm as a fixed starting position and allowed to move freely through the maze during a 5 min session. Between tests, the arms were thoroughly cleaned with 75% ethanol to eliminate any residual odors from previous mice. Alternation was defined as successive entries into the three arms on overlapping triplet sets. The number of total arm choices and the sequence of arm choices were recorded. The percent alternation was defined as the proportion of arm choices differing from the previous two choices and was calculated using the following equation: % alternations = (sequence of arm choices)/(total arm choices − 2) × 100 [25].

#### 2.1.6. Object Recognition Tests

Object recognition tests were also used to assess learning and memory in the experimental mice [26]. The probe test was performed on the third day after placing the mice into the chamber for 10 min to allow them to become familiar with the environment on the two days preceding the test. The mice were presented with two similar objects, A1 and A2, during the first session, and then, during the second session, A2 was replaced by a new object, B, of the same size and shape as object A2. Each mouse was allowed to explore the objects freely for 5 min during each session. Exploring behavior was considered as the mouse having its nose oriented toward the object and within about 2 cm from the object. The amount of time spent exploring each of the two objects was recorded and designated as EA and EB, respectively. Finally, the results were expressed as a discrimination index (DI), where DI = (EB − EA)/(EB + EA). The paths of mouse movement were tracked and analyzed using Any‐maze™ software (Shanghai XinRuan Information Technology Co., Ltd.).

### 2.2. Biochemical and Histochemical Analyses

#### 2.2.1. Sample Preparation

On the same morning at the end of the drug or control treatments, fecal samples (one or two pellets) from all mice were collected into Eppendorf tubes and rapidly frozen at −80°C. Blood was taken from the eye veins of the mice after the behavioral experiment and was allowed to stand for 30 min at RT and then centrifuged at 3500 rpm for 10 min. The supernatant was aspirated and stored at −80°C. After blood collection, the neck of each mouse was dislocated, and each head was rapidly removed, and half of the head was immobilized in 4% paraformaldehyde for 24 h at 4°C and then embedded in paraffin. Embedded brain tissue sections were continuously sliced into 4-5 mm sections for histological analysis. The other half of the head was divided into cortex and hippocampus on ice, rinsed, and stored at −80°C. The liver and small intestine tissue were, respectively, weighed to prepare 5% and 10% weight ratio tissue homogenates by adding normal saline. The homogenates were centrifuged at 3000 rpm for 10 min at 4°C, and then the supernatant was harvested.

#### 2.2.2. Hematoxylin-Eosin Staining

Mice were sacrificed after the behavioral experiment and the brain tissues were removed. The half-brain samples were immobilized in 4% paraformaldehyde for 24 h at 4°C and then embedded in paraffin. Embedded brain tissue sections were continuous sliced into 4-5 mm sections for hematoxylin-eosin staining according to the manufacturer's instructions (German Leica Instrument Co. Ltd.). The hippocampal CA1 area was observed and photographed under an optical microscope (BK-DM500, Chongqing Optec Instrument Co. Ltd., Chongqing, China).

#### 2.2.3. Immunohistochemistry

Immunohistochemistry was initiated by dewaxing the paraffin sections, and antigen retrieval was performed using boiling citrate buffer solution. This was followed by incubating the sections in a 3% H_2_O_2_ solution at RT for 10 min to block endogenous peroxidase activity. The brain tissues on these sections were then blocked by incubating with normal goat serum at RT for 15 min, and subsequently diluted antibodies (A*β*: 1:100 or* Escherichia coli*-LPS: 1:100; anti-A*β* 1-42 [ab39377] and anti-*E.* coli-LPS [ab35654] obtained from Abcam, Cambridge, UK) were added to brain tissues before incubating overnight at 4°C. Biotin-labeled secondary antibody was added and incubated for 15 min at RT, followed by incubation with streptavidin-biotin complex and 3,3′ diaminobenzidine chromogenic reagent. Finally, the sections were counterstained with hematoxylin and observed under a microscope. The integrated optical density (IOD) of the positively stained portions of each CA1 region was quantified using Image Pro Plus 6.0 software (Media Cybernetics, Rockville, MD).

#### 2.2.4. Western Blotting

Western blotting was used to detect the amount of AP present in tissues. The hippocampus was collected, lysed, and boiled at 100°C in 1:4 loading buffer and then centrifuged at 12,000 ×* g* for 10 min at 4°C. The supernatant was used to assay the protein levels. After electrophoresis (native polyacrylamide gel electrophoresis or sodium dodecyl sulfate-polyacrylamide gel electrophoresis), proteins were transferred to 0.2-*μ*M polyvinylidene fluoride (PVDF) membranes using a semidry transfer system. PVDF membranes with transferred proteins were incubated in blocking reagent for 1 h at RT and then incubated at 4°C overnight with primary antibody (rabbit anti-human TNSALP, 1:500; Beyotime Biotechnology, Shanghai, China). Membranes were next incubated with secondary antibody (AP-conjugated goat anti-rabbit IgG, 1:5000; BOSTER Biological Technology Co. Ltd, Wuhan, China) for 1.5 h at RT. Protein loading was detected using a super-enhanced chemiluminescence reagent (Applygen Technologies Inc., Beijing, China).

#### 2.2.5. Measurement of AP, LPS, and MDA

The levels of LPS in liver, serum, and feces were assessed with a quantitative chromogenic end-point Tachypleus amebocyte lysate assay kit (Xiamen Houshiji, Xiamen, China) according to the manufacturer's instructions. The levels of MDA in liver, serum, and small intestine were measured using a MDA assay kit (thiobarbituric acid method; Nanjing Jiancheng Bioengineering Institute, Nanjing, China), using an automatic chemical analyzer according to the manufacturer's instructions. Finally, the levels of AP enzyme in liver, serum, and small intestine were assessed using an AP assay kit (Nanjing Jiancheng Bioengineering Institute, Nanjing, China), according to the manufacturer's instructions.

#### 2.2.6. Quantification of Bacterial Populations by Quantitative Real-Time PCR

*(1) Fecal DNA Extraction and 16S rRNA Gene Sequencing*. DNA was extracted from mouse fecal samples (200–300 mg) using a TIANamp Stool DNA Kit (TianGen Biotech, Beijing, China) according to the manufacturer's instructions. To analyze changes in the populations of* E. coli* and* Lactobacillus*, which are representative Gram-negative bacteria and Gram-positive bacteria species in the gut microbiota, PCR and quality control of PCR products were carried out as described previously [27]. The primer sequences for amplifying the* 16S* rRNA gene from* Lactobacillus* (accession number: NZ_AYYW01000015) were 5′-GCCACATTGGGACTGAGACA-3′ (sense) and 5′-GGACAACGCTTGCCA CCTA-3′ (antisense). The primer sequences for amplifying the* 16S* rRNA gene from* E. coli* Esch (accession number: NC_000913.3) were 5′-GCGGACGGGTGAGTAATGT-3′ (sense) and 5′-CCCTCTTTGGTCTTGCGA-3′ (antisense). The different primers were also checked for their specificity using BLAST against the nucleotide database (NCBI).

*(2) Quantitative Real-Time PCR (qPCR)*. Standard curves were constructed using sequences from recombinant plasmids carrying the* 16S* rRNA genes of* E. coli* and* Lactobacillus*. The function describing the relationship between Ct (threshold cycle) and x (log copy number) for* Lactobacillus *was Ct = −3.4187x + 29.18, R^2^ = 1, and for* E. coli* was Ct = −3.0277x + 27.842, R^2^ = 0.99. Each reaction included 10 *μ*l Bestar® SybrGreen qPCR Mastermix (DBI® Bioscience, Shanghai, China), 0.5 *μ*l PCR Forward Primer (10 *μ*M), 0.5 *μ*l PCR Reverse Primer (10 *μ*M), 1 *μ*l DNA template, and 8 *μ*l ddH2O in real-time PCR amplification mixtures (20 *μ*l). Thermal cycling was performed using the Mx3000P detection system (Agilent Technologies Inc., State of California, America ) as follows: 40 cycles of 2 min at 95°C, 20 s at 95°C, 20 s at 58°C, and 20 s at 72°C. To determine the specificity of amplification, analyses of product melting curves were performed after the final cycle of each amplification at 94°C for 30 s, 65°C for 30 s, and 94°C for 30 s. Calypso software version 7.14 (http://cgenome.net/calypso/) was used to remove samples with fewer than 1000 sequence reads and 0.01% relative abundance across all samples. Reactions were repeated three times for each sample to achieve linearity.

### 2.3. Statistical Analyses

All data are expressed as the mean ± standard error of the mean (SEM). GraphPad Prism 6.0 (GraphPad Software, Inc., La Jolla, CA) was used to plot and analyze the data. Data between the two groups were compared using a Student's* t*-test. Comparison of data from multiple groups against one group was performed using one-way analysis of variance (ANOVA) followed by a* Dunnett's post hoc* test or a two-way repeated measures ANOVA with a* Tukey's multiple comparisons test*. The data from orientation and navigation tests were processed using a repeated measures univariate ANOVA with a general linear model using SPSS statistical software (SPSS Inc., Chicago, IL). A P value less than 0.05 was considered statistically significant.

## 3. Results

### 3.1. DART-TOFMS Analysis of the Main Components of FSS

A total of 37 putative active substances was detected in FSS in positive and negative ion modes. Ligustilide was the main putative active substance detected, and 31 other putative active substances with mass response intensities greater than 5000 (Table 2) were detected. Table 2 shows putative active substances detected in FSS by DART-TOFMS analysis.

### 3.2. FSS Rescues Cognitive Deficits in APP/PS1 AD Mice

**Figure 1:**
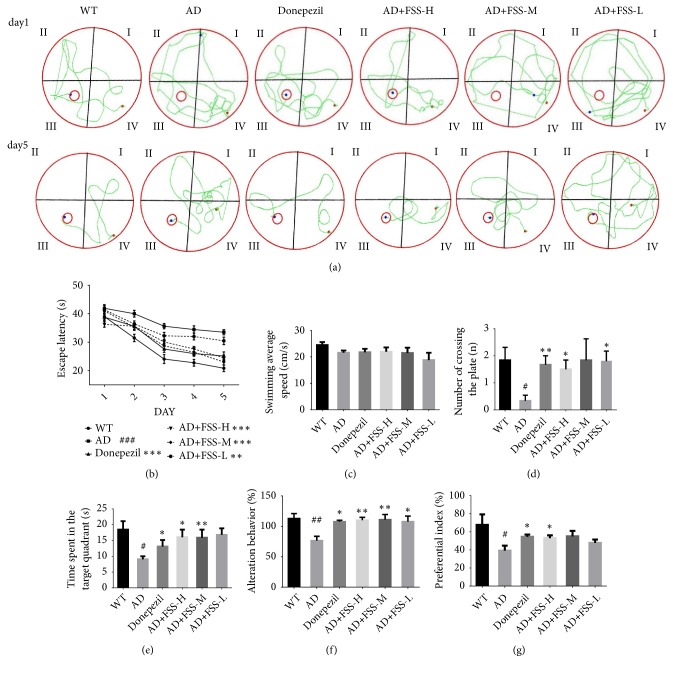
*FSS ameliorates cognitive dysfunction during behavioral testing in APP/PS1 AD mice*. (a) Total swimming paths of the respective groups on the first and fifth day. (b) Escape latency for five consecutive daily tests (n = 8 per group). (c) Swimming average speed in the probe trial. (d) Number of times mice swam across the target platform in the probe trial. (e) Time spent in the target quadrant in the probe trial. (f) Percentage alternation in the Y maze test. (g) Preferential index in the novel object recognition test. Data represent the mean ± SEM (n = 6 per group). # P < 0.05, ## P < 0.01, and ### P < 0.001 compared with WT. *∗* P < 0.05, *∗∗* P < 0.01, and *∗∗∗* P < 0.001 compared with AD. FSS: Fo Shou San; AD: Alzheimer's disease; WT: wild type; FSS-L: 1.6 g/kg/day; FSS-M: 3.2 g/kg/day; FSS-H: 6.4 g/kg/day.

As shown in Figure 1, the Morris water maze test demonstrated the effect of FSS on cognitive deficit in APP/PS1 mice. The total swimming paths were markedly longer in the AD mice compared with the WT control group (Figure 1(a)). The change in the length of the latency period in the water for each group was similar to the change observed for the total swimming distance (Figure 1(b)). On day 6, we conducted the probe trial with the platform removed, allowing the mice to swim freely in order to estimate their spatial working memory. The average swimming speed was similar among all groups (Figure 1(c)), but the AD mice spent less time in the target quadrant and swam across the plate fewer times than did those in the WT group (Figures 1(d) and 1(e)). After FSS treatment, the total lengths of the swimming paths for the FSS-H mice were shorter than those for the AD mice, and both the number of times the mice swam across the platform and the time spent in the target quadrant were greater than for the AD mice. However, the trend for the number of times the mice swam across the plate was not dose-dependent, perhaps due to individual differences that resulted in error. Also, there might have been a system error in the time spent in the target quadrant in the FSS-L mice, leading to longer times compared with those of the other two FSS treatment groups. Cognitive ability was also determined as the percent of alternation in the Y maze. The results showed that the AD mice had a lower percentage of alternation than did the WT group (Figure 1(f)). After FSS treatment, the percentage of alternation increased significantly, especially in the FSS-H and FSS-M mice, and this effect was superior to that in the donepezil-treated control group. The novel object recognition test evaluated the object recognition memory of the experimental mice. The preferential index was higher in the FSS-treated mice than in the AD mice (Figure 1(g)), but only the FSS-H mice had a significantly increased (P < 0.05) preferential index.

### 3.3. FSS Attenuates Neuronal Damage in the Hippocampus of APP/PS1 AD Mice

**Figure 2:**
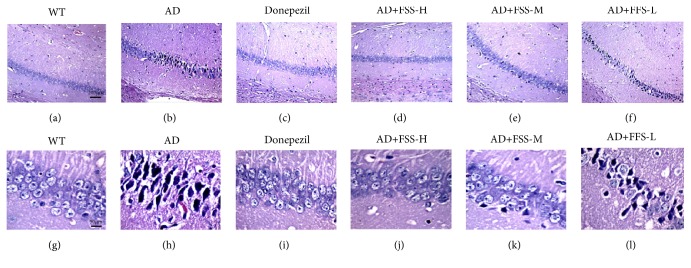
*FSS attenuates neuronal damage in the hippocampus of APP/PS1 AD mice*. The hippocampal CA1 regions of mice were observed using hematoxylin-eosin staining: at 100× (a–f) and 400× (g–l) magnification. (a, g) The WT group exhibited normal neuron structure, number, and nerve fibers. (b, h) The AD group exhibited loss of neurons, shrunken darkly stained cells, no peripheral staining, and severe distortion of neuron fibers. (c, i) The CA1 regions of the donepezil-treated group appeared to be very similar to those of the WT group. (d, j) and (e, k) The FSS-H group and FSS-M group both showed some cellular damage compared with the WT group. (f, l) The CA1 regions of the FSS-L group showed more damage, but less than that observed in the AD group. FSS: Fo Shou San; AD: Alzheimer's disease; WT: wild type; FSS-L: 1.6 g/kg/day; FSS-M: 3.2 g/kg/day; FSS-H: 6.4 g/kg/day.

Memory decline in AD is mostly limited to episodic memory, for which the hippocampus plays a crucial role. The mice hippocampal CA1 region was observed using hematoxylin-eosin staining (Figure 2) to analyze the effect of FSS on neuronal injury in APP/PS1 mice. The results showed that most neurons were lost or shrunken and darkly stained, with no peripheral staining and with severe distortion of neuron fibers in the CA1 areas of the hippocampus in the AD mice compared with that in the control WT mice. After FSS treatment, apart from the positive control mice that were treated with donepezil, the neuronal damage apparent in the FSS-M and FSS-H mice was attenuated, while the FSS-L mice exhibited significant neuronal damage.

### 3.4. FSS Attenuates LPS Levels in the Gut, Liver, Serum, and Brain of APP/PS1 AD Mice

**Figure 3:**
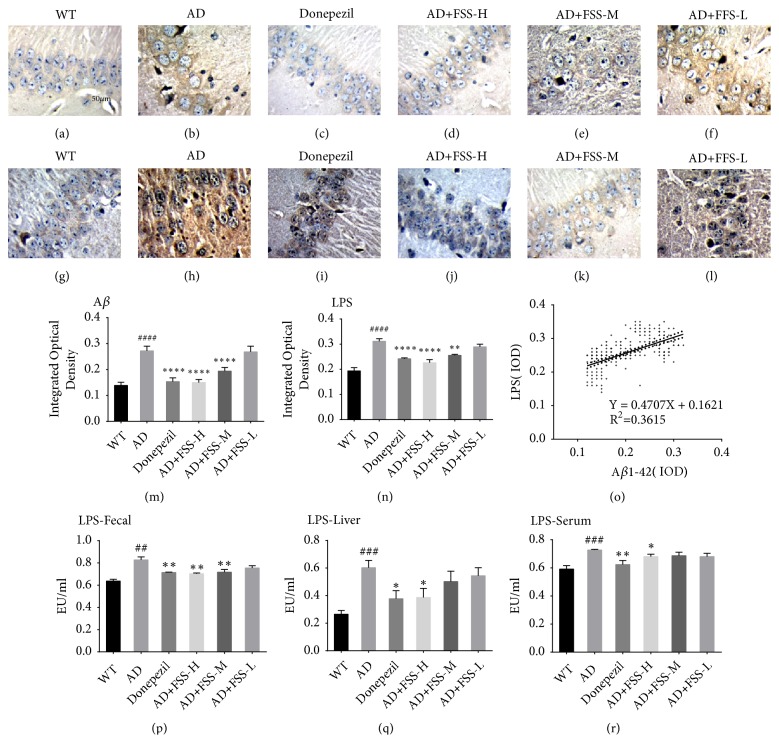
*FSS attenuates Aβ1-42 and LPS in the gut, liver, serum, and brain of APP/PS1 AD mice*. (a–l) Mouse hippocampal CA1 regions were observed at 400× magnification after immunohistochemical staining. (a–f) Expression levels of A*β*1-42. (g–l) Expression levels of LPS. (m, n) Graphs of the IOD of A*β*1-42 and LPS, respectively. (o) Correlation analysis between the IOD of A*β*1-42 and LPS. (p, q, r) Levels of LPS in the fecal matter, liver, and serum, respectively. Five photographs were taken of each area. IPP6.0 Pathology Image software was used to determine the IOD in each set of images, which was taken as the IOD value of the sample. Data represent the mean ± SEM (n = 8 per group). # P < 0.05, ## P < 0.01, ### P < 0.001 compared with WT. *∗* P < 0.05, *∗∗* P < 0.01, *∗∗∗* P < 0.001, compared with AD. FSS: Fo Shou San; AD: Alzheimer's disease; WT: wild type; FSS-L: 1.6 g/kg/day; FSS-M: 3.2 g/kg/day; FSS-H: 6.4 g/kg/day; A*β*1-42: amyloid-*β*; LPS: lipopolysaccharide; IOD, integrated optical density.

LPS exhibit the strongest induction of proinflammatory signaling in human brain cells [22]. To examine the effect of FSS on the amount of LPS in the brain, fecal matter, liver, and serum, immunohistochemical analysis was performed to quantify the expression levels of A*β*1-42 and LPS in the hippocampus. A quantitative chromogenic end-point Tachypleus amebocyte lysate assay kit (Xiamen Houshiji) was used to test the levels of LPS in the fecal matter, liver, and serum. The mean area covered by A*β*1-42 plaques and LPS in the hippocampus of the AD mice were both significantly higher than in that of the control WT mice (Figures 3(a), 3(b), 3(g), and 3(h)). Additionally, the levels of LPS were significantly higher in the fecal matter, liver, and serum of AD mice compared with that of the control WT mice (Figures 3(p), 3(q), and 3(r)). Then, we calculated the IOD of the positively stained portions of the hippocampus (Figures 3(m) and 3(n)). After FSS treatment the IOD of A*β*1-42 and LPS and the levels of LPS in the FSS-M and FSS-H mice and in the positive control mice treated with donepezil were significantly lower compared with those of the AD mice (Figures 3(m), 3(n), and 3(a)–3(l)). However, there was no noticeable decrease in the IOD of A*β*1-42 or LPS or in LPS levels in the FSS-L mice. In addition, the IOD of LPS showed a significant positive correlation with that of A*β*1-42 (r = 0.36, P < 0.0001) (Figure 3(o)).

### 3.5. FSS Regulates the Expression and Activity of Alkaline Phosphatase in Gut, Liver, Serum, and Brain of APP/PS1 AD Mice

**Figure 4:**
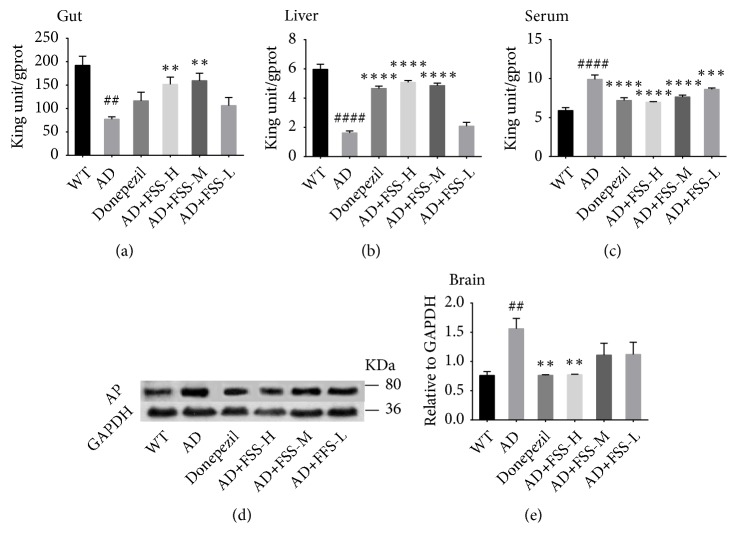
*FSS regulates expression and activity of AP in the gut, liver, serum, and brain of APP/PS1 AD mice*. (a) Quantification of AP in the gut (n = 7 per group). (b) Quantification of AP in the liver (except WT group, n = 9; n = 8 per group). (c) Quantification of AP in the serum (except WT and AD group, n = 7; n = 6 per group). (d) Expression of AP protein from hippocampus tissue (n = 3) detected by western blot analysis and quantified in (e) compared with expression of GAPDH (36 kDa) as the control. Data represent the mean ± SEM. ## p < 0.05 and ### p < 0.001 compared with WT. *∗* P < 0.05, *∗∗* P < 0.01, and *∗∗∗* P < 0.001 compared with AD. FSS: Fo Shou San; AD: Alzheimer's disease; WT: wild type; FSS-L: 1.6 g/kg/day; FSS-M: 3.2 g/kg/day; FSS-H: 6.4 g/kg/day; AP: alkaline phosphatase.

AP might play a role in the neuronal loss seen in AD. Western blots were performed to detect AP activity in the brain, and an AP assay kit was used to test AP activity in the gut, liver, and serum. AP activity was significantly higher in brain and serum but significantly lower in the gut and liver of the AD mice (Figure 4). After FSS treatment, AP activity in the brain and serum of the FSS-H mice and in the serum of the FSS-M mice were lower than in the AD mice (Figure 4). In contrast, the AP activity in the gut and liver of the FSS-H mice and FSS-M mice were higher than in the AD mice (Figure 4). However, changes in AP activity in the gut, liver, serum, and brain of the FSS-L mice were not particularly noticeable (Figure 4).

### 3.6. FSS Attenuates Lipid Peroxidation in Gut, Liver, Serum, and Brain of APP/PS1 AD Mice

**Figure 5:**
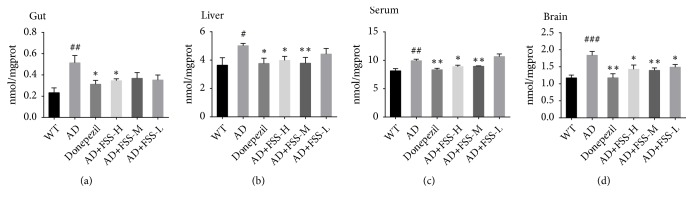
*FSS attenuates MDA in gut, liver, serum, and brain of APP/PS1 AD mice.* (a) Quantification of MDA in the gut (n = 7, 8, 7, 9, 8, 8 each group separately). (b) Quantification of MDA in liver (n = 6 per group). (c) Quantification of MDA in serum (except WT and AD group, n = 7; n = 6 per group). (d) Quantification of MDA in brain (n = 10, 10, 8, 10, 8, 9 each group separately). Data represent the mean ± SEM. ## P < 0.05 and ### P < 0.001 compared with WT. *∗* P < 0.05, *∗∗* P < 0.01, and *∗∗∗* P < 0.001 compared with AD. FSS: Fo Shou San; AD: Alzheimer's disease; WT: wild type; FSS-L: 1.6 g/kg/day; FSS-M: 3.2 g/kg/day; FSS-H: 6.4 g/kg/day; MDA: lipid peroxidation products.

MDA is a lipid peroxidation product and therefore serves as a biomarker for oxidative stress. To determine the effect of FSS on the oxidative stress status of experimental mice, we tested the levels of MDA in the gut, liver, serum, and brain using an MDA assay kit. As shown in Figure 5, the levels of MDA were significantly higher in the gut, liver, serum, and brain of the AD mice than in those of the control WT mice. However, MDA decreased sharply in the mice treated with 8-week oral administration of FSS compared with the AD mice. Except for gut in the FSS-M mice, the levels of MDA in FSS-M mice and especially in FSS-H mice decreased in liver, serum, and brain. However, the levels of MDA decreased only in the brain of FSS-L mice and were only slightly lower in the gut, liver, and serum under this low-dose treatment.

### 3.7. FSS Regulates Gut Bacteria in APP/PS1 AD Mice

**Figure 6:**
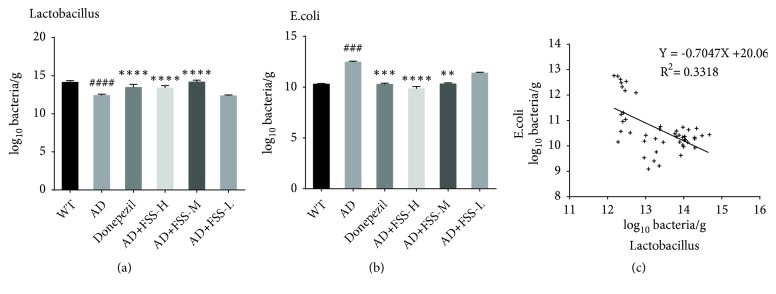
*FSS regulates populations of gut bacteria in APP/PS1 AD mice*. (a) Log_10_ bacteria copy number/g of* Lactobacillus*. (b) Log_10_ bacteria copy number/g of* E. coli*. (c) Correlation analysis between the copy numbers of* Lactobacillus* and* E. coli* measured by qPCR as log_10_ bacteria copy numbers/g in fecal samples collected from mice. Data represent the mean ± SEM (n = 8 per group). ## P <0.05 and ### P < 0.001 compared with WT. *∗* P < 0.05, *∗∗* P < 0.01, and *∗∗∗* P < 0.001 compared with AD. FSS: Fo Shou San; AD: Alzheimer's disease; WT: wild type; FSS-L: 1.6 g/kg/day; FSS-M: 3.2 g/kg/day; FSS-H: 6.4 g/kg/day.

Populations of* E. coli* and* Lactobacillus* were quantified by qPCR (Figure 6). Compared with the control WT mice, the amount of* Lactobacillus* was lower in the AD mice and increased after FSS treatment, but did not increase in the FSS-L mice (Figure 6(a)). However, the amount of* E. coli* was much higher in the AD mice compared with the control WT mice and fell after FSS treatment (Figure 6(b)). In addition, the amounts of* E. coli* and* Lactobacillus* were negatively correlated (Figure 6(c), r^2^ = 0.3318, P < 0.0001).

### 3.8. AP, LPS, and MDA Values Showed Significant Correlations in Gut, Liver, Serum, and Brain of APP/PS1 AD Mice

**Figure 7:**
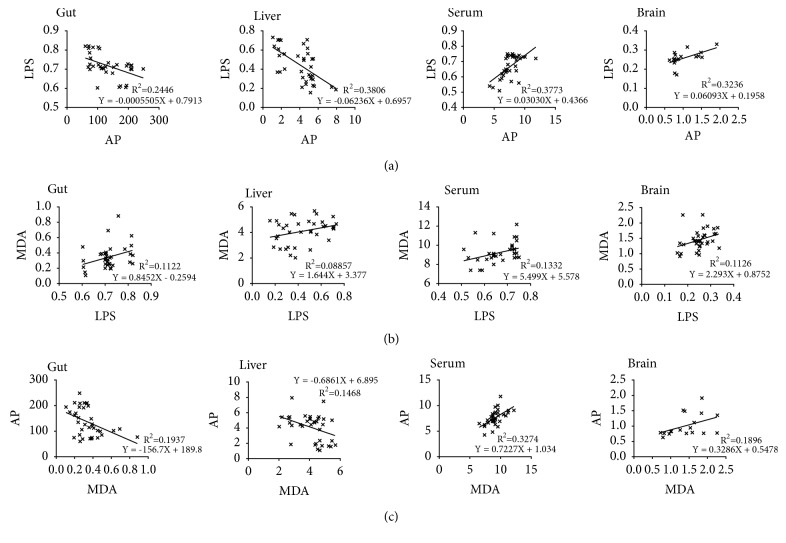
*Levels of AP, LPS, and MDA are significantly correlated in gut, liver, serum, and brain of APP/PS1 AD mice*. (a) Correlation analysis between AP and LPS levels in gut, liver, serum, and brain. (b) Correlation analysis between LPS and MDA levels in gut, liver, serum, and brain. (c) Correlation analysis between MDA and AP levels in gut, liver, serum, and brain. FSS: Fo Shou San; AD: Alzheimer's disease; AP: alkaline phosphatase; LPS: lipopolysaccharides; MDA: lipid peroxidation products.

The amounts of AP and LPS in gut and liver both showed significant negative correlations (r^2^ = 0.2446, P = 0.0022; r^2^ = 0.3806, p <0.0001) (Figure 7(a)) and in serum showed a significant positive correlation (r^2^ = 0.3773, P < 0.0001) (Figure 7(a)). The expression of IOD value of AP relative to that of GAPDH and the IOD of LPS in brain showed a significant positive correlation (r^2^ = 0.3236, P = 0.0138) (Figure 7(a)). The levels of LPS and of MDA in gut, serum, and brain all showed significant positive correlations (r^2^ = 0.1122, P = 0.0458; r^2^ = 0.1332, P = 0.0286 r^2^ = 0.1126, and P = 0.0455, respectively) (Figure 7(b)) and in liver showed a slight positive correlation (P = 0.0779) (Figure 7(b)). The levels of MDA and AP in gut and liver both showed significant negative correlations (r^2^ = 0.1937, P = 0.0072; r^2^ = 0.1468, p = 0.0211) (Figure 7(c)), in serum showed a significant positive correlation (r^2^ = 0.3274, P = 0.0003) (Figure 7(c)), and in brain showed a slight positive correlation (P = 0.0709) (Figure 7(c)).

## 4. Discussion

**Figure 8:**
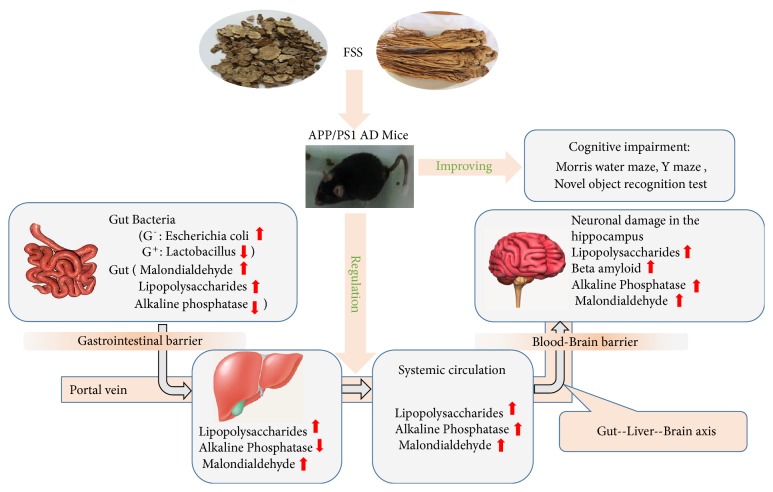
*Diagram illustrating a model for how FSS might attenuate Alzheimer's disease-related pathologies via the gut-liver-brain axis in APP/PS1 AD mice*. FSS might operate via the gut-liver-brain axis to regulate AP activity and gut bacterial populations, attenuate A*β*, LPS-related systemic inflammation, and oxidative stress (MDA), thereby alleviating AD-related pathologies in APP/PS1 AD mice. We hypothesize that FSS might improve the gastrointestinal (GI) barrier and blood-brain barrier, thereby ameliorating AD-related pathologies. A*β*: amyloid-*β*; AP: alkaline phosphatase; LPS: lipopolysaccharides; MDA: malondialdehyde, lipid peroxidation products; AD: Alzheimer's disease.

Our experiments involved 8-week oral administration of FSS to 6-month-old male APP/PS1 mice. Results indicated that FSS could ameliorate learning and memory impairments and prevent neuronal injury in APP/PS1 transgenic mice that exhibit an early-onset AD-like pathology via the gut-liver-brain axis by inhibiting lipid peroxidation, decreasing levels of LPS, regulating AP activity, and regulating gut bacteria populations (Figure 8).

AD is a very complex disease caused by a complicated interaction among genetic and environmental factors [28]. Most studies of AD involve A*β* or Tau pathologies [29] or neuroinflammation [30, 31]. Current AD drugs target cholinergic and glutamatergic neurotransmission and thus ameliorate symptoms. However, the long-term efficacy of these drugs in clinical practice remains controversial [2, 3].

Our study analyzed the effects of the well-known ancient paired-herb decoction FSS in an AD mouse model. After FSS treatment, we found that high and medium doses of FSS (6.4 and 3.2 g/kg/day, respectively) could ameliorate learning and memory abilities and prevent neuronal injury in APP/PS1 transgenic mice but that a low dose (1.6g/kg/day) was not therapeutic.

The gut microbiome has previously been associated with neuroinflammation and AD. An analysis of selected gut microbiota from the stool revealed an increase in* E. coli *populations in AD patients [32] and a decrease in occupancy of the gut by* Lactobacillus* in the Tg-APP/PS1 mouse model of AD [33].

We found that* E. coli* populations were higher in APP/PS1 transgenic mice. LPS found in the wall of Gram-negative bacteria could play a role in causing sporadic AD [17]. When populations of Gram-negative bacteria increase, highly proinflammatory neurotoxins such as amyloid and LPS can be released and pass through damaged GI tracts. These noxious molecules from the GI tract can then enter the systemic circulation system and possibly bypass the blood-brain barrier to enter the brain [34].

Furthermore, we found that* Lactobacillus* amounts were lower in APP/PS1 transgenic mice. Cow's milk fermented with* Lactobacillus *has attenuated LPS-induced memory deficit by increasing antioxidant enzymes (SOD, GSH, and GPx) and by decreasing MDA, AChE, and proinflammatory cytokines in mice [35].* Lactobacillus helveticus* could improve behavioral and cognitive impairments [36], and decreases in populations of these bacteria are correlated with AD pathology.

LPS have also been found in large amounts in brain lysates from the hippocampus and superior temporal lobe neocortex of AD brains [32, 33]. There is also evidence that blood LPS levels in AD patients are 3 times those in controls [14, 37], suggesting an association between LPS with AD.* E. coli* bacteria that bear LPS can trigger host inflammatory responses and induce the formation of extracellular amyloid plaques [14, 38, 39] that are very similar to the A*β* aggregates in the brains of AD patients [14, 40]. One report indicates that microbe-induced sources of amyloid in the GI tract, LPS, or other microbe-derived signaling molecules might contribute to both systemic and central nervous system amyloid burdens [14, 41]. The amyloid hypothesis suggests that accumulation of A*β* in the brain is critical to AD pathogenesis. A*β* exerts strong detrimental effects by inducing excitotoxicity [14, 42]. Because LPS and A*β* both act as agonists for the TLR4/CD14 receptor [14, 43, 44], they may establish a vicious circle in which LPS acts on the TLR4/CD14 receptor, thereby increasing A*β*, which in turn provides positive feedback to the TLR4/CD14 receptor, thus producing progressive damage in the brain, hippocampus, and cortical neurons, which are the most severely affected cells in AD [45].

We found not only that both A*β*1-42 and LPS quantities were higher in the hippocampus in our APP/PS1 mice, but also that LPS amounts were significantly and positively correlated with the levels of A*β*1-42 (r = 0.36, P < 0.0001). More importantly, we found that LPS was also higher in the fecal matter, liver, and serum of AD mice. This suggests that the gut-liver-brain axis in AD mice is abnormal. If so, LPS might cross from the GI tract into the liver, then enter the systemic circulation, and eventually cross blood-brain barrier into the brain.

LPS participates in the pathology of AD in two main ways. One is by strong induction of proinflammatory signaling, including several different chemokines, cytokines, and A*β* peptides in human neuronal-glial cells in primary coculture [22, 23, 40]. Another way might be via induction of increased A*β* production, which exerts strong neurotoxic effects. LPS administration causes poor memory retention in mice during MWM and Y maze tests and results in marked oxidative stress [16].

MDA is a lipid peroxidation product that is a marker of oxidative stress [46], which can mediate damage to neurons and eventually lead to dramatic neuronal loss and cognitive dysfunction [47]. The brains of AD patients are exposed to oxidative stress during the progression of the disease [48]. In our study, we found that brain, gut, liver, and serum all showed high levels of MDA in AD mice compared with those of control WT mice, demonstrating that oxidative stress occurred not only in the brain but also in the gut, liver, and systemic circulation, possibly through the gut-liver-brain axis. This resulted in damage to their normal function.

Our study shows that AP activity was significantly higher in the brain and serum, whereas it was lower in the gut and liver of AD mice compared with that of control WT mice. A related analysis found that AP, LPS, and MDA showed a significant correlation in brain, gut, liver, serum of APP/PS1 mice.

AP is an enzyme that is widely distributed in the liver, gut, brain, and blood and is present in neuronal membranes and induces neuronal toxicity via dephosphorylation of Tau protein, a mechanism that could play a role in the neuronal loss seen in AD. AP activity is significantly increased in the brain in both the sporadic and familial forms of AD, and AP activity is also significantly increased in the plasma in AD patients [49], as observed in our study.

AP has also been implicated in protection against inflammation in multiple diseases and promotion of intestinal microbial populations through hydrolysis of extracellular ATP/ADP to AMP and adenosine [18, 19] and might have protective interactions with the bacteria that inhabit or invade the GI system [20]. In addition, AP can dephosphorylate the lipid A moiety of bacterial-derived LPS. AP has also been identified as a potential regulator of the composition of the intestinal microbiome, which is an evolutionarily conserved function [21].

In our study, we found that the activity of AP was lower in both the gut and liver of AD mice, but was higher in the brain and serum of AD mice. This phenomenon suggests that lower AP activity decreases the ability to counteract inflammation in the gut and liver, allowing Gram-negative bacteria to grow and release a large amount of LPS and amyloid from the intestine into the liver, which then enters systemic circulation through the liver and eventually reaches the brain. When LPS enters systemic circulation, it may induce a compensatory increase in the activity of AP in the brain and serum, in contrast to the gut and liver.

## 5. Conclusion

In conclusion, we found a significantly higher amount of highly proinflammatory LPS and MDA not only in the brain, but also in the gut, liver, and serum of APP/PS1 transgenic mice. This suggests that LPS and MDA might reach the brain via the gut-liver-brain axis and cause neuronal injury that influences the learning and memory abilities of AD mice.

In this study, we showed that administration of high and medium doses of FSS inhibits the proliferation of Gram-negative bacteria, thereby inhibiting mass release of LPS, and decreases lipid peroxidation but increases the levels of* Lactobacillus*, which can mitigate these harmful factors. We also showed that FSS ameliorated behavioral and cognitive impairments in AD mice that might be related to the gut-liver-brain axis. This effect could be related to the ability of FSS to reduce populations of harmful Gram-negative bacteria that produce large amounts of highly proinflammatory factors such as amyloid and LPS. At the same time, FSS could promote populations of* Lactobacilli* that ameliorate lipid peroxidation and thereby decrease the level of proinflammatory LPS to maintain the balance of the gut bacteria and improve the anti-inflammatory effects of AP in gut and liver.

Thus, LPS, MDA, AP, and gut bacteria together might be potential targets for prevention or treatment of AD. More importantly, because it can address multiple targets associated with AD and is safe and suitable for long-term use, FSS could be a very potent drug for the treatment of AD.

The GI tract barrier and blood-brain barrier might become significantly more permeable to small molecules in AD mice than in control WT mice. If so, FSS might markedly ameliorate the AD-related pathologies, possibly by improving function of the GI tract barrier and blood-brain barrier to inhibit proinflammatory factors from reaching the brain.

## Figures and Tables

**Table 1:** Herbal constituents of FSS.

Components	Ratio
Danggui (Angelica sinensis (Oliv.) Diels, radix)	3
Chuanxiong (Ligusticum chuanxiong hort, rhizome)	2

FSS, Fo Shou San.

**Table 2:** Putative active substances detected in FSS by DART-TOFMS analysis.

Number	Name	Adduct	Detected Mass	Intensity	Number	Name	Adduct	Detected Mass	Intensity
1	ligustilide	+H	191.11	4.00E+06	20	levistilide A	+H	381.21	2.00E+06
2	limonene	+H	137.13	1707	21	ferulic acid	+H	195.07	3.00E+05
3	3,7,7-trimethyl-11 -methylene-spiro [5,5] undec-2-ene	+H	137.13	1707	22	*α*-pinene	+H	137.13	1707
4	terpinene	+H	137.13	1707	23	Z-butylidenephthalide	+H	189.09	4.00E+05
5	3-carene	+H	137.13	1707	24	B-butylidenephthalide	+H	189.09	4.00E+05
6	butylidene phthalide	+H	189.09	4.00E+05	25	N-butylidenephthalide	+H	189.09	4.00E+05
7	Senkyuno	+H	191.11	4.00E+06	26	E-ligustilide	+H	191.11	4.00E+06
8	butylphthalide	+H	191.11	4.00E+06	27	Z-ligustilide	+H	191.11	4.00E+06
9	senkyunolide A	+H	193.12	4.00E+06	28	angelica P.E A	+H	191.11	4.00E+06
10	sankyunolide A	+H	193.12	4.00E+06	29	Z-6, 8,7,3-diligustilide	+H	191.11	4.00E+06
11	sankyunolide I	+H	209.12	4.00E+05	30	senkyunolide G	+H	209.12	4.00E+05
12	sankyunolide F	+H	225.11	89833	31	senkyunolide I	+H	225.11	89833
13	neocnidilide	+H	195.14	4.00E+05	32	senkyunolide H	+H	225.11	89833
14	4-hydroxy-3-bntyl phthalide	-H	249.15	14824	33	Z,Z-6,6,7,3,a-diligustilide	-H	339.16	11920
15	cubebene	+H	205.19	5282	34	5-hydroxymethylfurfural	+H	127.04	10643
16	eudesma-4, 11-dlene	+H	205.19	5282	35	anisic acid	+H	153.05	16692
17	spathulenol	+H	221.19	14238	36	vanillic acid	+H	169.05	5077
18	secaline	-H	249.08	9369	37	4-vinylguaiaco1	+H	151.08	7488
19	progesterone prog	-H	313.22	704					

## Data Availability

The data used to support the findings of this study are available from the corresponding author upon request.
